# Evidence for mitigation of coral bleaching by manganese

**DOI:** 10.1038/s41598-018-34994-4

**Published:** 2018-11-14

**Authors:** Tom Biscéré, Christine Ferrier-Pagès, Antoine Gilbert, Thomas Pichler, Fanny Houlbrèque

**Affiliations:** 1 IRD, ENTROPIE (UMR 9220), BP A5, 98848 Nouméa cedex, New-Caledonia France; 2 Ginger Soproner, BP 3583, 98846 Nouméa cedex, New-Caledonia France; 3 Sorbonne Université, Collège Doctoral, F-75005 Paris, France; 4 Centre Scientifique de Monaco, Marine department, ecophysiology, 8 quai Antoine 1er, 98000 Monaco, France; 5 Department of Geosciences, University of Bremen, 28359 Bremen, Germany

## Abstract

Unprecedented mass coral bleaching events due to global warming and overall seawater pollution have been observed worldwide over the last decades. Although metals are often considered as toxic substances for corals, some are essential at nanomolar concentrations for physiological processes such as photosynthesis and antioxidant defenses. This study was designed to elucidate, the individual and combined effects of nanomolar seawater enrichment in manganese (Mn) and iron (Fe), on the main physiological traits of *Stylophora pistillata*, maintained under normal growth and thermal stress conditions. We provide, for the first time, evidence that Mn is a key trace element for coral symbionts, enhancing cellular chlorophyll concentrations, photosynthetic efficiency and gross photosynthetic rates at ambient temperature. Our experiment also highlights the key role of Mn in increasing coral resistance to heat stress-induced bleaching. While Mn-enriched corals did not bleach and did not reduce their rates of photosynthesis and calcification, control corals experienced significant bleaching. On the contrary to Mn, Fe enrichment not only impaired calcification but induced significant bleaching. Such information is an important step towards a better understanding of the response of corals to seawater enrichment in metals. It can also explain, to some extent, species susceptibility to environmental stress.

## Introduction

Coral reefs are one of the most biodiverse, complex and productive ecosystems on Earth^1,2^. Over the last decades, rising sea surface temperatures, owing to global warming, have triggered unprecedented mass bleaching events, during which corals lose their symbiotic algae and then undergo nutrient starvation, decreased growth and possible mortality (*e*.*g*.)^3^. Thermal stress-induced bleaching is often due to an over-production of damaging reactive oxygen species (ROS)^4^. Levels of ROS within the coral tissue are usually regulated by antioxidant enzymes, such as superoxide dismutase (SOD), peroxidases and catalases^5–7^. These enzymes can either neutralize free radicals of ROS by accepting or donating electron(s) to eliminate the unpaired condition of the radical or may directly react with the radicals and destroy them, or make them less reactive^8^. During thermal stress, ROS exceed the enzymes’ ability to detoxify them, leading to the direct damage of proteins, lipids or DNA^4,9^.

In addition to global change-related disturbances, coral reefs have also to cope with the local deterioration of the seawater quality, due to increased pollution in inorganic and organic nutrients, particle loads and sedimentation^10^. Many reefs around the world (*e*.*g*. Costa Rica, Panama, Red Sea, Thailand, New Caledonia) are also exposed to elevated metal concentrations^11–17^, brought by urban storm water run-off, industrial effluents, mining-operations and atmospheric contaminants^18^. Experimental studies have emphasized the harmful effect of high concentrations of metals on coral reproduction and early life stages^19–24^. The only three studies which have focused on the combined effects of thermal stress and metal pollution (copper and nickel)^12,25,26^, have highlighted an increased bleaching susceptibility of adult corals under metal pollution^13,26^ and a reduced thermal tolerance of coral larvae^25^.

Although many metals, such as mercury^27^, copper^28^ or lead^21^, are toxic for living organisms, even at nanomolar concentrations, some of them can play key roles in the functionning of photosynthetic organisms (*e*.*g*. phytoplankton, plants, algae) (reviewed by^29^). For example, manganese (Mn) and iron (Fe), whose concentrations in the tropical and subtropical coastal seawaters can reach 10.8 *µ*g L^−1^ and 2.61 *µ*g L^−1^, respectively^30–33^, are key elements of photosynthetic molecules. Mn is indeed an essential component of the Oxygen Evolving Complex (OEC) of photosystem II, whereas Fe is needed for the structure of the chlorophyll, of the photosystems I and II, the cytochrome b6f complex, and the ferredoxin^34–36^. Both are also cofactors of the antioxidant enzymes, such as superoxide dismutase (MnSOD and FeSOD)^37,38^, which continuously scavenge ROS within the coral tissue as described above^37–40^. With the exception of a single coral study, showing that iron limitation increases coral bleaching susceptibility^41^, all others have been performed on plants or phytoplankton. They showed that metal limitation impairs the photosynthetic efficiency of photosystem II^34,42,43^ and enhances the oxidative load, by decreasing antioxidant enzymes’ activity and ROS consumption^42^.

The aim of this work was to investigate the individual and combined effects of different levels of manganese (Mn) and iron (Fe), on the main physiological traits of the scleractinian coral species *Stylophora pistillata*, maintained under ambient temperature and thermal stress conditions. The hypotheses were that i) natural, *in situ* concentrations of Fe and Mn are not a limiting factor for coral productivity under ambient temperature conditions; ii) Fe and Mn can become limiting nutrients for coral productivity due to an increase need of metals for the synthesis of antoxidant enzymes or for chlorophyll repair. We thus hypothesize that an enrichment in Mn and/or Fe can increase the coral resistance to bleaching; iii) we also hypothesize that a combined enrichment in manganese and iron has additive effects compared to an enrichment in either manganese or iron alone.

## Results

### Multivariate analysis

**Figure 1:**
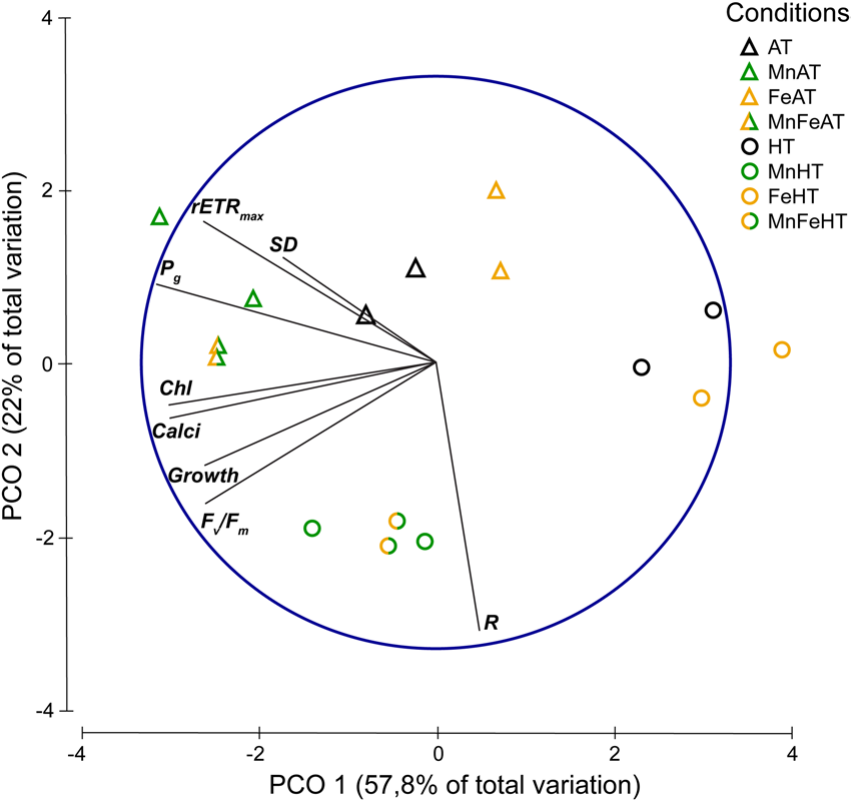
Principal Coordinate Analysis (PCoA) comparison of colony physiological traits maintained during six weeks either at 26 (AT) or 32 °C (HT) to ambient manganese and iron concentrations (0.06 and <0.22 *µ*g L^−1^); to higher manganese concentrations (4.1 *µ*g L^−1^) (Mn); to higher iron concentrations (3.0 *µ*g L^−1^) (Fe); and to both higher concentrations in manganese and iron (MnFe). SD, rETR_max_, P_g_, Chl, Calci, Growth, F_v_/F_m_ and R represent, respectively, the *Symbiodinium* density, the maximum electron transport rate, the gross photosynthetic rate, the chlorophyll concentration, the calcification rate, the growth rate, the photosynthetic efficiency and the respiration rate.

Principal Coordinate Analysis (PCoA) comparison of coral physiological traits maintained under the eight Mn-Fe-temperature conditions revealed a similar response between duplicated tanks (Fig. 1). PCoA yielded two principal components, PCO1 and PCO2 that explain 51% and 28% of the total variance respectively. The first component (PCO1), is defined by positive correlations between Mn enrichment and photosynthetic (P_g_, rETR_max_ and F_v_/F_m_, Chl concentrations) or growth parameters (calcification, growth rates) (r > 0.7). PCO1 also demonstrates that Fe enrichment at 32 °C has an opposite effect than manganese. The second component (PCO2) separates corals exposed to ambient and high temperatures and is defined by high weights from the “respiration” parameter (r > 0.7).

### Effect of thermal stress (32 °C) alone on control corals (not exposed to metal enrichment)

**Figure 2:**
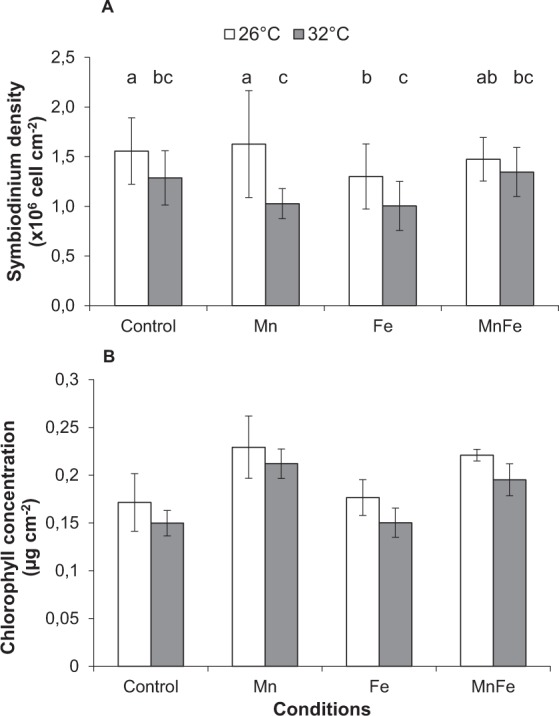
*Symbiodinium* density (**A**) and total chlorophyll concentration (**B**) of *Stylophora pistillata* (mean ± SD, n = 3) exposed during six weeks either at 26 or 32 °C to ambient manganese and iron concentrations (0.06 and < 0.22 *µ*g L^−1^) (Control); to higher manganese concentrations (4.1 *µ*g L^−1^) (Mn); to higher iron concentrations (3.0 *µ*g L^−1^) (Fe); and to both higher concentrations in manganese and iron (MnFe).

**Figure 3:**
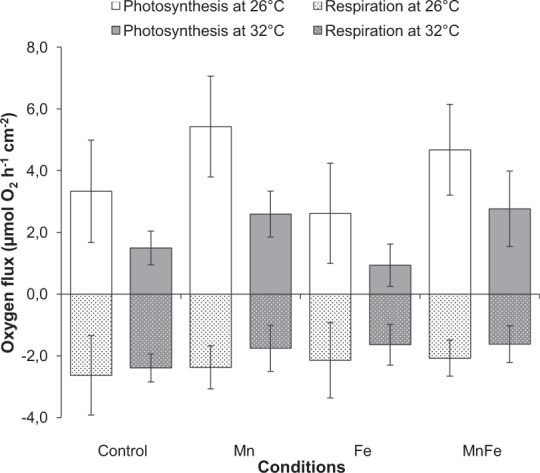
Photosynthetic and respiration rates of *Stylophora pistillata* (mean ± SD, n = 3) exposed during six weeks either at 26 or 32 °C to ambient manganese and iron concentrations (0.06 and <0.22 *µ*g L^−1^) (Control); to higher manganese concentrations (4.1 *µ*g L^−1^) (Mn); to higher iron concentrations (3.0 *µ*g L^−1^) (Fe); and to both higher concentrations in manganese and iron (MnFe).

**Figure 4:**
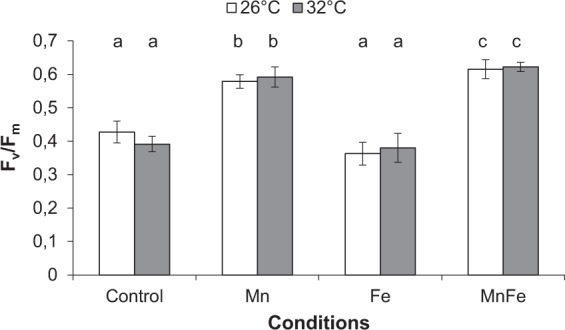
*Stylophora pistillata* photosynthetic efficiency (F_v_/F_m_) (mean ± SD, n = 5) exposed during six weeks either at 26 or 32 °C to ambient manganese and iron concentrations (0.06 and <0.22 *µ*g L^−1^) (Control); to higher manganese concentrations (4.1 *µ*g L^−1^) (Mn); to higher iron concentrations (3.0 *µ*g L^−1^) (Fe); and to both higher concentrations in manganese and iron (MnFe).

**Figure 5:**
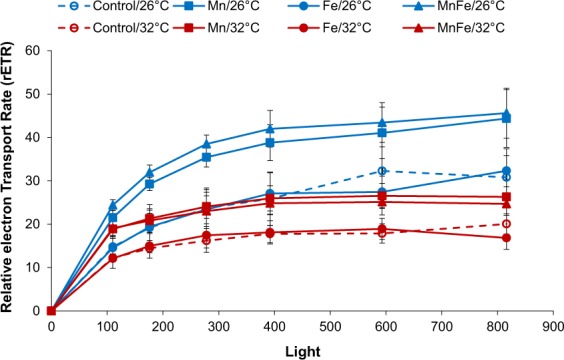
Relative electron transport rates (rETR) of *Stylophora pistillata* (mean ± SD, n = 5) exposed during six weeks either at 26 or 32 °C to ambient manganese and iron concentrations (0.06 and <0.22 *µ*g L^−1^) (Control); to higher manganese concentrations (4.1 *µ*g L^−1^) (Mn); to higher iron concentrations (3.0 *µ*g L^−1^) (Fe); and to both higher concentrations in manganese and iron (MnFe).

**Figure 6:**
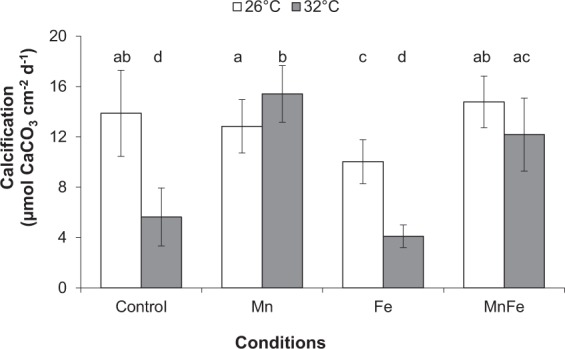
*Stylophora pistillata* calcification rates (mean ± SD, n = 3) exposed during six weeks either at 26 or 32 °C to ambient manganese and iron concentrations (0.06 and <0.22 *µ*g L^−1^) (Control); to higher manganese concentrations (4.1 *µ*g L^−1^) (Mn); to higher iron concentrations (3.0 *µ*g L^−1^) (Fe); and to both higher concentrations in manganese and iron (MnFe).

**Figure 7:**
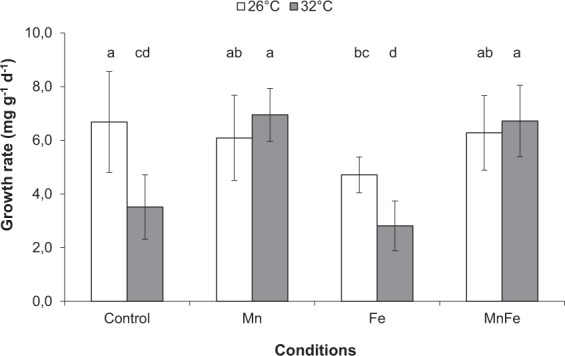
*Stylophora pistillata* growth rates (mean ± SD, n = 5) exposed during six weeks either at 26 or 32 °C to ambient manganese and iron concentrations (0.06 and <0.22 *µ*g L^−1^) (Control); to higher manganese concentrations (4.1 *µ*g L^−1^) (Mn); to higher iron concentrations (3.0 *µ*g L^−1^) (Fe); and to both higher concentrations in manganese and iron (MnFe).

As expected, thermal stress decreased symbiont density, chlorophyll content and Pg by 18%, 11% and 56%, respectively, compared to 26 °C (ANOVA; p < 0.001; Figs 2A,B and 3). No thermal stress effect was observed on the F_v_/F_m_ (ANOVA; p > 0.05; Fig. 4), but reduced rETR_max_ by 41% (ANOVA; p < 0.001; Fig. 5), respiration rates by 20% in all conditions (ANOVA; p < 0.05; Fig. 3), calcification and growth rates by 59% and 47% respectively (LSD test; p < 0.001; Figs 6 and 7).

### Combined effect of iron enrichment and thermal stress

At 26 °C, Fe enrichment decreased symbiont density by 11% compared to the control condition (LSD test; p < 0.05; Fig. 2A), but had no effect on chl content, P_g_ and respiration rates, as well as on the F_v_/F_m_ and rETR_max_ (ANOVA; p > 0.05; Figs 2B, 3–5). Nevertheless, calcification and growth rates were decreased by ca. 28% compared to the control condition (LSD test; p < 0.01; Figs 6 and 7). No interactive effect between temperature and Fe enrichment was observed (ANOVA; p > 0.05). At 32 °C and under Fe enrichment, thermal stress reduced symbiont density by 35%, P_g_ rates and rETR_max_ by 65% and 45% respectively compared to those of Fe corals at 26 °C (ANOVA; p < 0.001; Figs 2A, 3 and 5). As well, thermal stress reduced growth and calcification rates by 40% and 59% respectively compared to those of Fe corals at 26 °C (LSD test; p < 0.001; Figs 6 and 7).

### Combined effect of manganese or manganese-iron enrichment and thermal stress

At 26 °C, no effect of Mn or Mn-Fe enrichment was observed on symbiont density (Fig. 2A) and on the calcification and growth rates (LSD test; p > 0.05; Figs 6 and 7). However, both enrichments stimulated chlorophyll concentrations by 30%, as well as P_g_ and rETR_max_ by 69% and 29% compared to the controls at 26 °C (ANOVA; p < 0.001; Figs 2B, 3 and 5). F_v_/F_m_ was also enhanced by ca. 40% (LSD test; p < 0.001; Fig. 4).

No interactive effect was observed between temperature and Mn or Mn-Fe enrichment on the tissue parameters, P_g_ or respiration rates and the F_v_/F_m_ (ANOVA; p > 0.05, Figs 2A,B, 3 and 4). Although chlorophyll concentrations were stimulated by 36% in Mn enriched colonies at 32 °C compared to the ones of control corals at 26 °C (ANOVA; p < 0.001; Fig. 2B), thermal stress reduced the symbiont density by 34% and 13% in Mn and MnFe corals, P_g_ by 52% and 41% and rETR_max_ by 39% and 44% compared to those of control corals at 26 °C (ANOVA; p < 0.001; Figs 2A, 3 and 5). However, P_g_ and rETR_max_ remained significantly higher (29% and ca. 79%, respectively) than those of control corals submitted to the same thermal stress (ANOVA; p < 0.001; Figs 3 and 5). Finally, a significant interaction was observed between Mn and temperature on calcification and growth rates (ANOVA; p < 0.01, Figs 6 and 7). Both rates were enhanced by thermal stress and Mn addition compared to control corals (LDS test; p < 0.001). Mn enriched colonies at high temperature maintained the same calcification and growth rates than at 26 °C (LSD test; p > 0.05). At 32 °C, Fe however interacted with Mn enrichment as MnFe enriched corals decreased their calcification rates by 21% compared to those of Mn enriched corals under high temperature (LSD test; p < 0.05).

## Discussion

While massive bleaching events intensify around the world, there is a need to identify the environmental factors, which can increase the resistance and resilience of corals to thermal-stress induced bleaching. Here, we provide the first evidence that Mn is a key trace element for the coral - *Symbiodinium* association. It directly enhances symbiont photosynthesis, and indirectly host metabolism, since symbionts translocate most of their photosynthates to the animal host. Mn thus mitigates the negative impact of short-term thermal stress on coral bleaching. We have also observed that seawater enrichment with 3 *µ*g Fe L^−1^ induced a decrease in calcification rates and counteracts the positive effects of Mn on coral bleaching. Finally, we did not find any link in this study between rates of photosynthesis and calcification, suggesting that inorganic carbon may be a limiting factor for corals under manganese enrichment.

Results obtained in this study first clearly show that Mn is a limiting nutrient for coral photosynthesis, at natural seawater concentrations (0.06 *µ*g Mn L^−1^) such as those measured outside New Caledonia’s barrier reef^30^. Photosynthetic efficiency (F_v_/F_m_, rETR_max_) as well as chlorophyll concentrations and gross photosynthetic rates were all enhanced under 4 *µ*g Mn L^−1^ enrichment, suggesting that corals have high requirements in Mn. Although such Mn stimulation has been previously documented for phytoplankton photosynthesis^44^, it was unknown for *Symbiodinium in hospite*. Manganese is involved in several photosynthetic processes, which overall explain the higher rates of carbon fixation observed under normal growth conditions. This metal enters into the composition of the oxygen-evolving complex (OEC) of photosystem II^45–47^, which splits water molecules into electrons, protons and oxygen and shuttles the electrons into the photosystem II reaction center. Mn is also directly involved in chlorophyll synthesis, via the isoprenoid biosynthetic pathway, as demonstrated in phytoplankton and plants^48–50^.

Our experiment also highlights the key role of Mn in increasing coral resistance to heat stress-induced bleaching. At high temperature, Mn-enriched corals presented higher chlorophyll concentrations, photosynthetic and calcification rates than control corals, which experienced significant bleaching, likely due to high oxidative stress^5,51^. In addition to enter into the composition of photosynthetic molecules, Mn is a key component of anti-oxidant enzymes, such as manganese superoxide dismutase (MnSOD)^52^. This metallo-enzyme, located in *Symbiodinum*^53,54^, is used to eliminate ROS, which are continuously produced through photosynthesis^4^. SOD thus protects the whole symbiotic association from photosynthetic and thermal stress-induced ROS, and thereby avoids chlorophyll degradation^55,56^ (as observed in the present study) and likely DNA damage, protein unfolding and lipid peroxidation^4,9^. Moreover, when Mn replace zinc at the active site of the carbonic anhydrase molecule, this later shows a peroxidase activity with bicarbonate-dependent activity^57,58^. As such, it can detoxify the H_2_O_2_ produced during thermal stress inside the coral cells. Our results therefore show that bleaching is delayed by at least two weeks in presence of manganese but the long-term effect of manganese on the bleaching susceptibility of corals remains to be tested.

Although Mn enrichment allowed corals to maintain maximal rates of photosynthesis and calcification during thermal stress, we did not observe in this study a coupling between photosynthesis and calcification. Following the stimulation of photosynthesis, we could have expected to observe an increase in the rates of short- and/or long-term calcification. Such correlation between photosynthesis and calcification is well known^59^. Calcification rates of scleractinian corals are indeed enhanced in the light compared to the dark and this process is called light enhanced calcification (reviewed by^59^). This can be primarily attributed to the photosynthetic activity of the symbionts, which has several impacts, such as the provision of essential molecules for the organic matrix synthesis or the elevation of pH at the site of calcification^59–61^. A link between photosynthesis and calcification has been observed with nickel enrichment, another trace element, which stimulated calcification in *Pocillopora damicornis* and *Acropora muricata* directly and indirectly through the stimulation of photosynthesis^62^. The lack of calcification enhancement in manganese-enriched corals at 26 °C could be explained by a potential limitation in dissolved inorganic carbon (DIC) or a competition for DIC between photosynthesis and calcification, as already observed in previous experiments involving nutrient, iron or cobalt enrichments^12,63–65^. Overall, corals living in a DIC scarce environment may experience DIC limitation and invest a great deal of energy into concentrating carbon at the photosynthesis site^66^.

Varying effects of iron exposure on corals have been observed in the literature and seem to depend on its concentration in seawater. As iron is involved in metabolic processes such as nitrogen fixation, antioxidant defenses, photosynthetic electron transfer or chlorophyll biosynthesis^7,38,56^, a complete lack of iron in seawater was shown to induce coral bleaching^41^. A slight enrichment (0.28 *µ*g L^−1^) enhanced photosynthesis, but significantly decreased calcification, likely due to a competition between these two processes for dissolved inorganic carbon (DIC^65^). The highest enrichment used in this study (3 *µ*g L^−1^), comparable to concentrations measured in some reefs along the New Caledonia’s coast^30^, not only impaired calcification, but induced significant bleaching. Wells *et al*.^36^ showed that the intracellular ROS production within *S*. *pistillata* endosymbionts was increased when exposed to Fe-enriched seawater. Such increased ROS level can explain the bleaching observed in this experiment and the subsequent decrease in calcification rate. Indeed, the loss of symbionts induces starvation and decreased energy, which is essential for calcification.

Two interesting interactions between iron and manganese enrichment were observed in this study. A synergetic interaction between Mn and Fe enrichments, which stimulated the photosynthetic efficiency, has been recorded. This observation suggests that *Symbiodinium* photosynthesis is more dependent on the quantity of available electron brought by the O_2_ evolving complex (Mn-dependent) than by molecules ensuring the transfer of these electrons (Fe-dependent), since an enrichment only in Fe does not modify the rates of photosynthesis. In addition, there was a strong negative effect of Fe on coral calcification, since Fe enrichment partially inhibited the positive effect of manganese on calcification rates under thermal stress.

**Figure 8:**
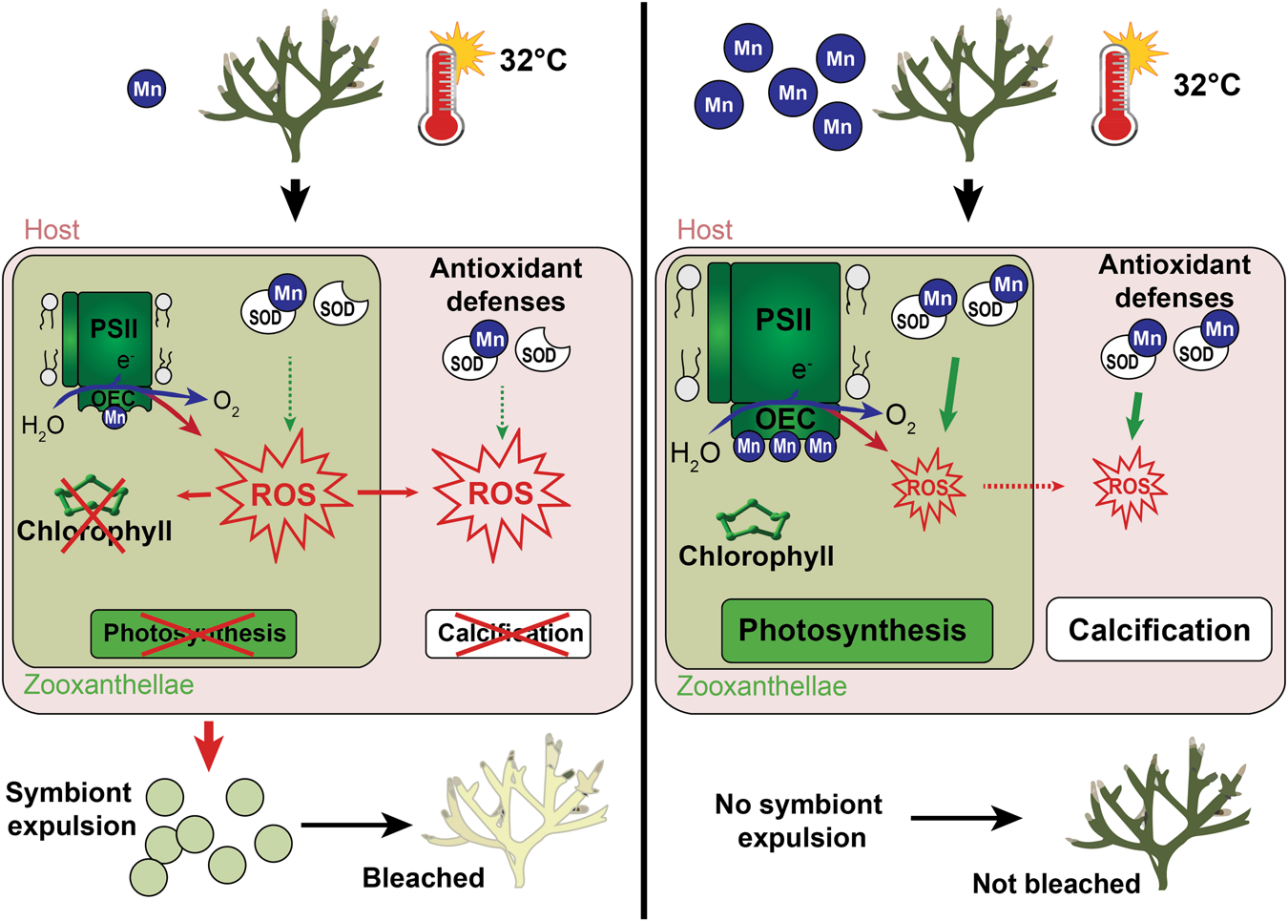
Schematic diagram summarizing the effects of a manganese enrichment on adult coral physiology under ambient temperature or thermal stress. Mn increases coral resistance to heat stress-induced bleaching by boosting all physiological parameters compared to control corals, which experienced significant bleaching likely due to oxidative stress. The fact that (1) Mn enters into the composition of the oxygen-evolving complex (OEC) of photosystem II, (2) Mn is directly involved in chlorophyll synthesis and (3) Mn is also a key component of anti-oxidant enzymes, such as Mn superoxide dismutase (MnSOD) (used to eliminate ROS during thermal stress), justify the stimulating properties of this metal.

In summary, our paper highlights the importance of taking into account seawater metal concentrations to explain the heat-stress induced changes in coral physiology. Overall, Mn appeared as a limiting nutrient for coral photosynthesis and antioxidant capacity. Mn enrichment thus increased rates of photosynthesis and calcification, and decreased the bleaching susceptibility of *S*. *pistillata* (Fig. 8). According to our results, high Mn concentrations in seawater can potentially explain the between-reef variability in bleaching susceptibility. Different uptake or assimilation rates of Mn between coral species can also explain species-specific bleaching. For example, reefs in New Caledonia, which have the specificity to be located in Mn-enriched waters, experienced their first massive bleaching event only in February 2016. However, the degree heating weeks (DHW) had yet already exceeded 8 °C-Weeks (threshold values normally leading to widespread bleaching and mortality) several times in the past (in 1980, 1996, 2005) without causing coral bleaching (https://coralreefwatch.noaa.gov/satellite/vs/melanesia.php#Amedee_NewCaledonia). More in-depth studies are however needed to confirm or rule out this hypothesis. In addition, this study has explored short-term exposure time to Mn enrichment, and/or to thermal stress. More studies are needed to refine the role of Mn enrichment in the mitigation of coral bleaching. Indeed, responses of corals are highly dynamic and tend to change with increasing accumulation of stress; the beneficial role of Mn on symbiont’s photosynthesis may therefore change with the length or the amplitude of the stress. Otherwise, it has also to be noticed that metal pollution is never based on a unique metal enrichment, and we clearly showed that metal interactions have to be considered when assessing the response of corals to thermal stress. Fe enrichment significantly impaired coral calcification, through processes that remain to be investigated. Finally, in the future, reefs will also experience increased ocean acidification, which not only will impact corals directly, but also indirectly through changes in the metal water chemistry^67^. Additional experiments, simultaneously testing the effects of acidification and warming on manganese uptake rates are thus needed to provide a better understanding of the metal role in the limitation of coral bleaching phenomenon.

## Methods

### Coral collection and experimental setup

Effects of manganese and iron enrichment alone or together associated with a thermal stress on coral physiology were assessed on the scleractinian coral, *Stylophora pistillata*, originating from the Red Sea and grown in aquaria at the Centre Scientifique de Monaco. To perform this experiment, 384 apex (2-cm long) (i.e. microcolonies) were sampled from six large mother colonies. Microcolonies were then hung on nylon wires, evenly distributed in 16 tanks (25 L) before the three weeks recovering period under controlled conditions. To improve recovery, corals received nauplii of *Artemia salina* twice a week. Tanks were supplied with filtered seawater pumped from 40 meters depth, renewed at a rate of 12 L h^−1^. Temperature (26 or 32 ± 0.2 °C) was controlled all along the experiment by heaters connected to Elli-Well PC 902/T controllers. Corals was illuminated by eight 400 W metal halide lamps (HPIT, Philips) at an irradiance of 200 ± 10 *µ*mol photons m^−2^ s^−1^ (photoperiod was 12 h:12 h light:dark). Once a week, light was controlled by a LI-COR data logger (LI- 1000) connected to a spherical quantum sensor (LI-193).

After the recovery, eight experimental conditions in a cross factor design (with manganese, iron and temperature as factors) were tested in duplicate. In other words, 4 metal conditions under two temperatures (26 °C and 32 °C) were achieved in duplicate: 1) control (C), with 0.06 ± 0.05 *μ*g Mn L^−1 ^^68^ and < 0.22 *μ*g Fe L^−1 ^^65^, 2) manganese enrichment (4.1 ± 0.75 *μ*g Mn L^−1^), 3) iron enrichment (3.00 ± 0.2 *µ*g Fe L^−1^) and 4) manganese-iron enrichment at the two above concentrations (MnFe) (Supplementary Fig. S1 and Table 1). Concentrations were chosen to be ecologically relevant and correspond to the highest concentration measured along the coast of New-Caledonia^30^.

Two peristaltic pumps (ISMATEC) ensured the metal enrichments by continuously supplying the experimental tanks with 15 mL h^−1^ of a solution of stable manganese (MnCl_2_, Humeau, France) or stable iron (FeCl_2_, Humeau, France). In order to avoid any concentration gradient in the tank and to ensure a good seawater brewing, tanks were equipped with a submersible pump (Aquarium system, micro-jet MC 320, Mentor, OH, USA). After three weeks under metal enrichment, temperature was gradually increased during one week up to 32 °C (1 °C per day) in two tanks of each metal condition and maintained for an additional two weeks without stopping metal enrichment. Photosynthetic efficiency, growth rates, calcification rates, and tissue parameters (zooxanthellae density, chlorophyll and protein concentration) were measured at the beginning of the experiment, after three weeks of metal enrichment and at the end of the two weeks of thermal stress.

Metals enrichment were realized with their divalent forms, Fe^2+^ and Mn^2+^, because they are the most soluble form of these metals and are considered as the most bioavailable^69,70^. While Mn^2+^ remains largely bioavailable in the time due to its very long half-life time^71^, the half-life of the Fe^2+^ ions is only of few minutes at 30 °C^72,73^. Thus, in order to be sure that the concentrations in the tanks match with the concentrations desired, seawater samples have been realized in each tank to measure metal concentrations. Samples were filtered using a 0.2 µm filter and were acidified with 1% of ultrapure concentrated nitritic acid (HNO_3_^−^), before sending for analysis (see supplementary materials for values).

Concentrations of Mn and Fe were determined by sector field inductively coupled plasma-mass spectrometry (SF-ICP-MS) using a Thermo Element 2 instrument. Mass spectra were collected in medium resolution and MeOH was added to enhance ionization. Based on repeat measurements, as well as external and internal standards analytical uncertainty was estimated to be better than 5%. All reagents used were analytical grade quality or better.

### Photosynthetic parameters

All the photosynthetic parameters were measured as described in Biscéré *et al*.^62^. Thus, photosynthetic efficiency (F_v_/F_m_) of the coral symbiont and the relative electron transport rate (rETR) of their Photosystem II (PSII) were firstly measured early in the morning with a DIVING-PAM fluorometer (Walz, Germany) (*n* = 5 for each tank). Then, immediately after, respiration (R) and gross photosynthesis rates (Pg) were assessed on three apex from each tank under their own living conditions (temperature, metal enrichment, light). Finally, samples were frozen at −20 °C to further estimate their *Symbiodinium* density and chlorophyll concentrations. Respiration and gross photosynthesis rates, as well as *Symbiodinium* density and chlorophyll concentration were normalized per unit surface area (cm²). This one was estimated by weighting apex before and after dipping in hot paraffin. The weight of paraffin gave the surface area thanks to a linear regression of known surfaces against paraffin weight^74^.

### Growth parameters

As for photosynthetic parameters, methods to measure growth and calcification rates are fully described in Biscéré *et al*.^62^. Long-term calcification was assessed using the buoyant weight technique^75^, when short-term calcification rates were estimated using the alkalinity anomaly technique^76^. Five microcolonies in each tank were weekly weighted all along the experiment to measure the long-term calcification, which was calculated as the daily change in dry weight and expressed in mg g^−1^ d^−1^. However, only three microcolonies were used to estimate short-term calcification rates at each time because data being expressed as *µ*mol CaCO_3_ cm^−2^ d^−1^, samples needed to be dried immediately after to measure their surface area.

### Statistical analysis

Principal Coordinate Analysis (PCoA) was performed on a multidimensional similarity matrix based on the Euclidian distance between all experimental conditions, using normalized parameters (F_v_/F_m_, ETR, growth rates, calcification rates, photosynthetic and respiration rates, symbiodinium and chlorophyll concentrations). PCoA was optimized with vector overlays of raw Pearson correlations (limited to r > 0.6). All similarity analyses were performed using PRIMER 6 statistical software^77^.

Three-way ANOVAs were used to test the effects of manganese and iron concentrations, thermal stress and their interactions on the 8 coral physiological parameters assessed. All the statistical results are given in the Supplementary Table 1. Data were first tested for normality and homoscedasticity using Shapiro Wilk’s test and Bartlett’s test respectively. For normality requirements, all data were transformed according to the Box-Cox transformation^78^, except for respiration rates, for which the square root transformation was applied. When the ANOVA determined significant differences between factors, a post-hoc pair-wise Least Significant Difference (LSD) test was performed to assign differences to specific factors. Individual differences revealed by the LSD test are represented by letters on the figures. All data are expressed as mean ± SD. All tests were performed using R software.

## Electronic supplementary material

Supplementary information on the materials and methods and the statistical results
