# Supplementary material for: Evidence for mitigation of coral bleaching by manganese
Source: Sci Rep. 2018 Nov 14;8:16789. doi: 10.1038/s41598-018-34994-4 (PMC6235957; doi:10.1038/s41598-018-34994-4)
Supplement: Supplementary file 1 — Supplementary information on the materials and methods and the statistical results [file 41598_2018_34994_MOESM1_ESM.docx]

**Evidence for mitigation of coral bleaching by manganese**

Biscéré T.^1,2,3,*^, Ferrier-Pagès C.^4^, Gilbert A.^2^, Pichler T.^5^, Houlbrèque F.^1^

**Fig. S1**. Experimental set up with eight conditions in a crossed factor design. Every condition was in duplicate. Control (Ctrl): ambient concentrations of manganese and iron; Manganese (Mn): higher manganese concentrations; Iron (Fe): higher iron concentrations; Manganese/Iron (MnFe): higher manganese and iron concentrations. Seawater parameters are shown below each condition. Mn and Fe are in µg L^-1^; Conductivity in mV; DIC in µmol L^-1^; O_2_ in mg L^-1^.


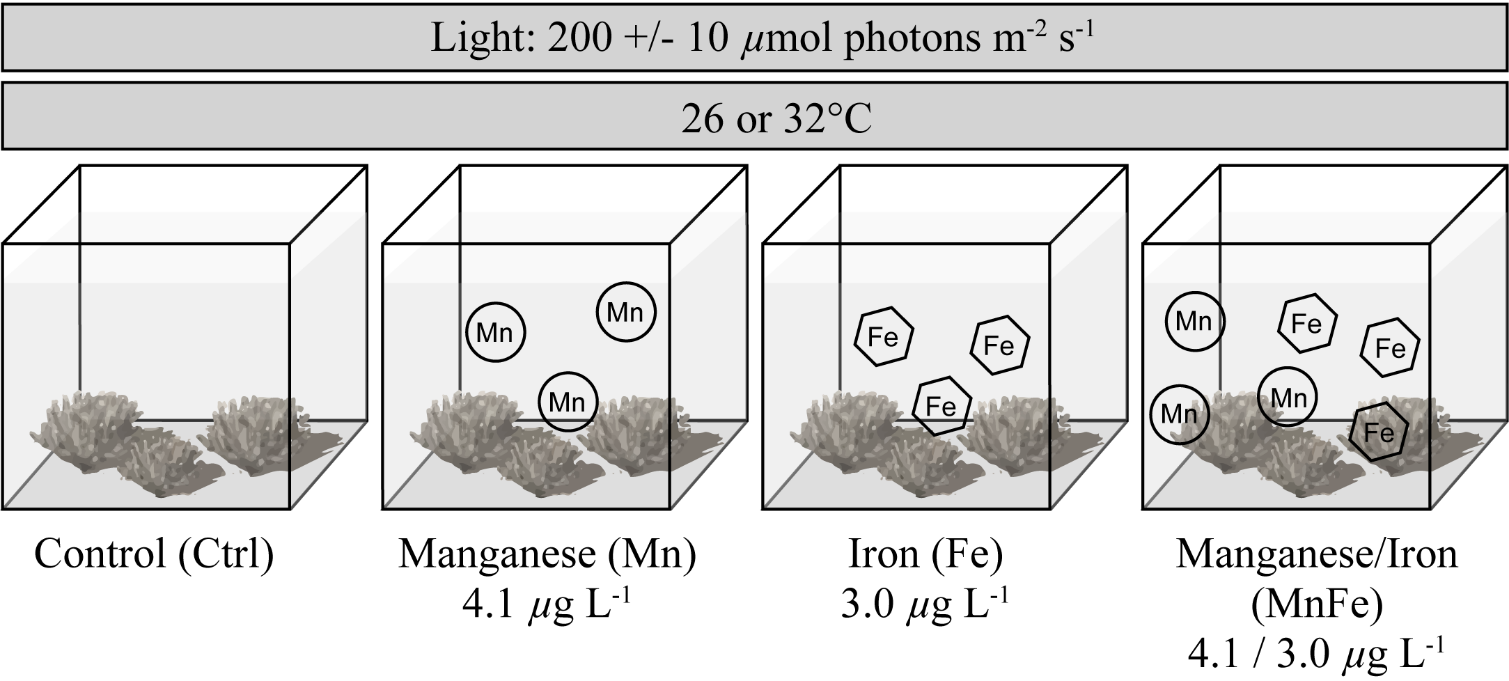


**Control (Ctrl)**

Mn: 0.06 ± 0.05

Fe: <0.22

pH: 8.05 ± 0.03

Conductivity: -66.1

DIC: 2226.4 ± 49.3

O_2_: 6.82 ± 0.02

Salinity: 37.3 ± 0.2

**Manganese (Mn)**

Mn: 4.1 ± 0.75

Fe: <0.22

pH: 8.03 ± 0.02

Conductivity: -65.2

DIC: 2230.1 ± 41.2

O_2_: 6.48 ± 0.04

Salinity: 37.2 ± 0.2

**Iron (Fe)**

Mn: 0.06 ± 0.05

Fe: 3.0 ± 0.3

pH: 8.03 ± 0.03

Conductivity: -65.2

DIC: 2221.1 ± 31.9

O_2_: 6.49 ± 0.07

Salinity: 37.1 ± 0.3

**Manganese/Iron (MnFe)**

Mn: 4.1 ± 0.75

Fe: 3.0 ± 0.3

pH: 8.07 ± 0.05

Conductivity: -68

DIC: 2243.5 ± 38.2

O_2_: 7.17 ± 0.02

Salinity: 37.1 ± 0.1

**Table 1.** Metal concentrations in each incubation condition at T_0_, T_0_ + 3 weeks and T_0_ + 6 weeks. Ctrl: Control; Mn: Manganese enriched; Fe: Iron enriched; MnFe: Both manganese and iron enriched.

|  | **Conditions** | | | | | | | |
| --- | --- | --- | --- | --- | --- | --- | --- | --- |
| **Time** | Ctrl | | Mn | | Fe | | MnFe | |
|  | Mn | Fe | Mn | Fe | Mn | Fe | Mn | Fe |
| **T_0_** | <3 | <2 | 4.9 | <2 | <3 | 2.5 | 4.4 | 3.0 |
| **T_0_ + 3 weeks** | <3 | <2 | 3.9 | <2 | <3 | 3.1 | 4.1 | 3.4 |
| **T_0_ + 6 weeks** | <3 | <2 | 3.8 | <2 | <3 | 3.6 | 3.9 | 2.7 |

**Table 2.** Summary of three-way ANOVAs testing the combined effect of manganese (Mn) (ambient: 0.06 *µ*g L^-1^ and enriched: 4.1 *µ*g L^-1^) and iron concentrations (Fe) (ambient: <0.22 *µ*g L^-1^ and enriched: 3.0 *µ*g L^-1^) and thermal stress (Temp) (ambient: 26°C and warm: 32°C) on *Stylophora pistillata* physiological parameters during the 6-weeks experiment.

|  | *Stylophora pistillata* | | | | |
| --- | --- | --- | --- | --- | --- |
| Source of variation | | SS | df | F-ratio | *p*-Values |
| *Symbiodinium*.cm^-2^ | |  |  |  |  |
| Mn | | 1.02x10^11^ | 1 | 1.057 | 0.310 |
| Fe | | 8.12x10^10^ | 1 | 0.842 | 0.365 |
| Temp | | 1.21x10^12^ | 1 | 12.479 | 0.001** |
| Mn x Fe | | 3.70x10^11^ | 1 | 3.831 | 0.01* |
| Mn x Temp | | 1.89x10^10^ | 1 | 0.196 | 0.660 |
| Fe x Temp | | 1.53x10^11^ | 1 | 1.581 | 0.216 |
| Mn x Fe x Temp | | 1.81x10^11^ | 1 | 1.878 | 0.178 |
| Error | | 3.77x10^12^ | 40 |  |  |
| Total chlorophyll.cm^-2^ | |  |  |  |  |
| Mn | | 3.2x10^-2^ | 1 | 79.786 | 0.000*** |
| Fe | | 2.89x10^-4^ | 1 | 0.700 | 0.407 |
| Temp | | 6.21x10^-3^ | 1 | 15.087 | 0.000*** |
| Mn x Fe | | 7.12x10^-4^ | 1 | 1.728 | 0.196 |
| Mn x Temp | | 1.8x10^-5^ | 1 | 0.043 | 0.836 |
| Fe x Temp | | 1.26x10^-4^ | 1 | 0.306 | 0.582 |
| Mn x Fe x Temp | | 1.1x10^-5^ | 1 | 0.027 | 0.870 |
| Error | | 1.6x10^-2^ | 40 |  |  |
| Gross photosynthesis | |  |  |  |  |
| Mn | | 37.483 | 1 | 20.590 | 0.000*** |
| Fe | | 2.588 | 1 | 1.421 | 0.240 |
| Temp | | 51.141 | 1 | 28.093 | 0.000*** |
| Mn x Fe | | 0.363 | 1 | 0.199 | 0.657 |
| Mn x Temp | | 1.129 | 1 | 0.620 | 0.435 |
| Fe x Temp | | 0.881 | 1 | 0.484 | 0.490 |
| Mn x Fe x Temp | | 0.441 | 1 | 0.242 | 0.625 |
| Error | | 72.817 | 40 |  |  |
| Respiration | |  |  |  |  |
| Mn | | 0.718 | 1 | 0.882 | 0.353 |
| Fe | | 2.086 | 1 | 2.564 | 0.117 |
| Temp | | 2.447 | 1 | 3.007 | 0.032* |
| Mn x Fe | | 0.483 | 1 | 0.593 | 0.445 |
| Mn x Temp | | 0.079 | 1 | 0.096 | 0.757 |
| Fe x Temp | | 0.007 | 1 | 0.009 | 0.924 |
| Mn x Fe x Temp | | 0.134 | 1 | 0.164 | 0.687 |
| Error | | 32.546 | 40 |  |  |
| F_v_/F_m_ | |  |  |  |  |
| Mn | | 0.831 | 1 | 511.831 | 0.000*** |
| Fe | | 0.0005 | 1 | 0.332 | 0.565 |
| Temp | | 0.0009 | 1 | 0.611 | 0.436 |
| Mn x Fe | | 0.015 | 1 | 9.820 | 0.002** |
| Mn x Temp | | 0.006 | 1 | 3.725 | 0.057 |
| Fe x Temp | | 0.0004 | 1 | 0.243 | 0.623 |
| Mn x Fe x Temp | | 0.0011 | 1 | 0.673 | 0.414 |
| Error | | 0.117 | 72 |  |  |
| rETR_max_ | |  |  |  |  |
| Mn | | 1301.7 | 1 | 27.621 | 0.000*** |
| Fe | | 5.1 | 1 | 0.107 | 0.744 |
| Temp | | 5901.3 | 1 | 125.224 | 0.000*** |
| Mn x Fe | | 1 | 1 | 0.022 | 0.882 |
| Mn x Temp | | 89.7 | 1 | 1.902 | 0.172 |
| Fe x Temp | | 23.7 | 1 | 0.501 | 0.480 |
| Mn x Fe x Temp | | 3.5 | 1 | 0.074 | 0.786 |
| Error | | 3393.1 | 72 |  |  |
| Calcification rates | |  |  |  |  |
| Mn | | 348.7 | 1 | 64.779 | 0.000*** |
| Fe | | 33.34 | 1 | 6.194 | 0.017* |
| Temp | | 150.67 | 1 | 27.990 | 0.000*** |
| Mn x Fe | | 12.44 | 1 | 2.310 | 0.136 |
| Mn x Temp | | 149.95 | 1 | 27.856 | 0.000*** |
| Fe x Temp | | 6.18 | 1 | 1.147 | 0.290 |
| Mn x Fe x Temp | | 7.25 | 1 | 1.384 | 0.005** |
| Error | | 215.32 | 40 |  |  |
| Growth rates | |  |  |  |  |
| Mn | | 93.027 | 1 | 53..011 | 0.000*** |
| Fe | | 11.515 | 1 | 6.561 | 0.012* |
| Temp | | 14.84 | 1 | 8.456 | 0.004** |
| Mn x Fe | | 6.653 | 1 | 3.790 | 0.027* |
| Mn x Temp | | 55.926 | 1 | 31.869 | 0.000*** |
| Fe x Temp | | 0.34 | 1 | 0.193 | 0.661 |
| Mn x Fe x Temp | | 5.04 | 1 | 2.872 | 0.094 |
| Error | | 126.349 | 72 |  |  |
